# High HIV Prevalence among Asylum Seekers Who Gave Birth in the Netherlands: A Nationwide Study Based on Antenatal HIV Tests

**DOI:** 10.1371/journal.pone.0134724

**Published:** 2015-08-21

**Authors:** Simone Goosen, Christian J. P. A. Hoebe, Quita Waldhober, Anton E. Kunst

**Affiliations:** 1 Department of Public Health, Academic Medical Center, University of Amsterdam, Amsterdam, The Netherlands; 2 Netherlands Association for Community Health Services, Utrecht, The Netherlands; 3 Department of Sexual Health, Infectious Diseases and Environmental Health, South Limburg Public Health Service, Geleen, The Netherlands; 4 Department of Medical Microbiology, School of Public Health and Primary Care (CAPHRI), Maastricht University Medical Centre (MUMC), Maastricht, The Netherlands; Public Health Agency of Barcelona, SPAIN

## Abstract

**Objectives:**

Asylum seekers are considered to be a particularly vulnerable group with respect to HIV. Data on the HIV prevalence among asylum seekers, however, are scarce. The aim of this study is to map the HIV prevalence among asylum seekers who gave birth in The Netherlands.

**Methods:**

We used a nationwide electronic medical records database from the community health services for asylum seekers (MOA). The study population consisted of 4,854 women and girls who delivered in asylum reception between 2000 and 2008. A unique electronic health data base was used and case allocation was based on ICPC-codes.

**Results:**

The number of women and girls that was HIV positive during their last pregnancy was 80, of which 79 originated from sub-Saharan Africa. The prevalence for women from this region of origin (3.4%) was high compared to women from all other regions of origin (0.04%; OR = 90.2; 95%CI 12.5–648.8). The highest HIV prevalence rates were found for women from Rwanda (17.0%) and Cameroon (13.2%). HIV prevalence rates were higher among women who arrived in reception without partner (OR = 1.82; 95%CI 0.75–4.44) and unaccompanied minors (OR = 2.59; 95%CI 0.79–8.49), compared to women who arrived in reception with partner.

**Conclusions:**

We conclude that, among asylum-seeking women from sub-Saharan Africa giving birth in The Netherlands, the HIV prevalence is high compared to the host population. For women from other regions of origin, the prevalence is at the same level as in the host population. The high HIV prevalence underlines the importance of preventive interventions and voluntary HIV testing for sub-Saharan African asylum seekers as from shortly after arrival.

## Introduction

There is a pressing need to tackle the public health challenge of HIV in Europe and migrants are a key population according to the European Action Plan for HIV/AIDS 2012–2015 of the World Health Organization (WHO) [1]. High HIV prevalence rates among migrants are attributed to a combination of the HIV epidemiology in the countries of origin, specific vulnerabilities associated with the migration process, and inequalities in access to HIV prevention and treatment in the host country [2–5].

For developing appropriate preventive and curative healthcare services, it is crucial to understand the distribution and the determinants of HIV/AIDS in Europe’s vulnerable migrant populations [6]. Very few European countries, though, have data on HIV prevalence among these groups [7]. The European Centre for Disease Prevention and Control (ECDC) recently stated that countries should improve the availability of HIV prevalence data on migrants, especially for groups that may be particularly vulnerable [8].

Asylum seekers are persons who have fled their country of origin, have applied for recognition as a refugee in another country, and are awaiting a decision on their application [9]. They constitute a growing population in Western host countries: the number of asylum requests in the EU-28 rose from just below 200 000 to almost 450 000 in 2013 [10]. Asylum seekers may particularly be affected by HIV as war and displacement may have resulted in reduced access to HIV prevention services, disruption of social networks, and increased exposure to sexual violence and sex in return for food and shelter [11]. Single women and especially unaccompanied minor asylum seekers (UMAs) are particularly vulnerable to unsafe sex [12].

Insight into the distribution of HIV among asylum seekers in European host countries is very limited and based on small studies only [4]. A study in the UK found that 11 out of 288 asylum seekers were HIV positive (3.9%) and in a study in Italy 8 out of 529 were HIV positive (1.5%) [13,14]. Health professionals in The Netherlands have expressed their concerns about the HIV prevalence and the risk behaviours among asylum seekers. However, for The Netherlands only data on AIDS related mortality are available. In the period 2002–2005 the age standardised mortality ratio (SMR) due to AIDS for asylum seekers compared to the general population was 14.0 for males (95%CI 6.8–25.9) and 40.0 for females (95%CI 14.7–87.1) [15].

The availability of HIV surveillance data for asylum seekers in European countries is limited, because most countries have not traditionally collected information about migrant status in their health information systems and because denominator data are often lacking [4]. A potential source of information is a unique database in The Netherlands that contains health data for all asylum seekers who arrived in The Netherlands between 2000 and 2008 [16,17]. In this database, accurate data on the HIV prevalence is available only for pregnant women as they are the only group systematically being offered an HIV test.

The aim of this study is to map the prevalence of HIV among asylum seekers who gave birth in The Netherlands. We will assess HIV prevalence rates according to age at the time of delivery, country of origin, family composition at arrival, and the migration phase at the time of conception.

## Methods

Asylum seekers in The Netherlands are provided accommodation in centres managed by the Central Agency for the Reception of asylum seekers (COA). They have similar healthcare entitlements as residents of The Netherlands [18]. From January 2000 until December 2008 COA contracted the Community Health Services for Asylum Seekers (MOA) for providing public health services for all asylum seekers in The Netherlands. MOA nurses and public health physicians worked in close collaboration with family practitioners who were contracted by a health insurance company. Family practitioners referred pregnant asylum seekers to mainstream midwives, who offered antenatal services in line with Dutch standards. For the delivery, asylum-seeking women are referred to hospital [19]. MOA offered all asylum seekers a non-mandatory health assessment within six weeks after arrival. HIV counselling and testing were offered in case of risk factors, e.g. sexual violence or paid sex. MOA also offered interventions aimed at preventing the transmission of HIV and Sexually Transmitted Infections (STIs).

The antenatal HIV screening policy in The Netherlands up to 2004 was aimed at women at increased risk of HIV, the so called targeted selective screening [20]. As from January 2004 the policy was changed into universal antenatal HIV testing [20,21]. This means that an HIV test is offered to all women at the first antenatal visit, which preferably takes place before the 13th week of pregnancy. Tests were offered according to the opt-out principle [20,21]. Between 2006 and 2008, the national participation rate for HIV screening was 99.8% [21]. No coverage data are available for asylum seekers. During the study period, HIV diagnosis in The Netherlands was based on an ELISA test, followed by an immunoblot or RNA test [21].

### Study population and case definition

The MOA database contains longitudinal demographic and reception data for all asylum seekers who arrived in asylum reception in The Netherlands during the period 2000–2008. The MOA database is derived from the main administrative system of the COA, which is the basis for the provision of housing and coverage of healthcare costs and included a dedicated part for medical purposes. It contains health care data of MOA and family practitioners for the period that the asylum seekers stayed in asylum reception [16]. The database also includes data for all newborns in asylum reception and their mothers.

MOA staff and family physicians used the problem-oriented record (POR) method for general medical recording [22]. In this method, main and chronic health problems are recorded on the problem list along with International Classification of Primary Care (ICPC) code, date of diagnosis, and a short open field description. MOA staff entered problem list data from paper medical records in the electronic medical record system. As of 2009 the organisation of health care for asylum seekers changed and the use of the nationwide database was discontinued.

We first selected all children born in asylum reception between 1 January 2000 and 31 December 2008. Next, we selected their mothers by using the social unit number, which allows linkage between family members. HIV case attribution started with identification of HIV status among women who delivered in reception on the basis of ICPC-code B90. The number of women with code B90 was 82. The consistency between the ICPC-code and the open field description was manually checked. For 7 women their medical record contained ICPC-code B90 but the open field texts referred to a negative HIV test without any further indication of HIV status. These women were *not* allocated case status. Next, an open field description search was done for the text ‘HIV’ or ‘AIDS’ for women without a B90 ICPC-code. For 6 women the open field description contained a clear description of HIV diagnosis. The total number of women with a record of HIV diagnosis was 81. For 75 of these women the date of HIV diagnosis was before the date of their last delivery in reception; they were allocated case status. For three women the date of HIV diagnosis was within two months after their last delivery; they were also allocated case status. For three other women, the time between delivery and recording of HIV status was longer. For two of these women, their new born was recorded to be HIV positive with a diagnosis date around the same date as their mother (4 and 7 months after delivery). As it is very likely that they were HIV positive at the time of delivery, these women were allocated case status. The third woman was diagnosed four years after delivery and was not allocated case status. For the newborn children, HIV case status allocation followed the same approach as for their mothers. We identified women who gave birth to more than one child during their stay in reception by linking all children born in reception to their mother and by counting the number of newborn children for each mother.

### Independent variables

The variable ‘age group at delivery’ refers to the age at the time of the last delivery in reception. The variable ‘family composition’ distinguishes three groups of women: women who arrived in asylum reception with a partner, women who arrived without a partner, and unaccompanied minor asylum seekers (UMAs; children who have been separated from both parents and relatives, and who are not being cared for by an adult) [23]. The variable ‘migration phase at time of conception’ is derived by combining the date of arrival with the date of delivery. It distinguishes between women who got pregnant before arrival, within the first year after arrival or more than one year after arrival in asylum reception.

‘Country of origin’ is the country recorded by the immigration department; in general the country of nationality. Only countries with 30 or more women in the study population were analysed individually. Countries were grouped into ‘Regions of origin’ following the World Bank classification [24].

The variable ‘HIV testing policy at first antenatal consultation’ distinguishes between pregnancies for which the first antenatal consultation took place before or after the implementation of universal antenatal HIV testing in The Netherlands [21,25]. This distinction corresponds to delivery before and after 1 July 2004, as the first consultation preferably takes place before the 13th week of a pregnancy.

### Analysis

The HIV prevalence rate was calculated as the number of women that were allocated case status, divided by the total number of women that delivered in reception. The mother-to-child-transmission (MTCT) rate was calculated as the number of newborns with HIV diagnosis divided by the number of women who were HIV positive at the time of delivery. HIV and MTCT prevalence rates are presented as percentages.

Binary logistic regression was used to calculate odds ratios (OR) with 95% confidence intervals (CI) that express how the likelihood of having HIV is associated with age group at delivery, family composition, country of origin, migration phase at time of conception and the HIV testing policy at first antenatal consultation. We used multivariate regression models to control for all other variables included in the analysis. Statistical Software SPSS (IBM Inc., Version 20.0, Somers, NY, USA) was used for all analyses.

### Ethics

In line with Dutch legislation, the MOA privacy statement included a statement on the use of data for epidemiological purposes. Unique identifying numbers in the database were encrypted. Because we used only data collected for health care purposes, the medical ethics review committee (METC) of the Academic Medical Center at the University of Amsterdam stated that no METC approval was required (letter W12-276#12.17.0315).

## Results

The study population consisted of 4,854 women who delivered during their stay in reception. Demographic characteristics of these women are presented in Table 1. The total number of women who were recorded to be HIV positive at the time of their last delivery in reception was 80. The overall HIV prevalence for asylum seekers at the time of the last delivery in reception was 1.6%. Seventy-nine of the HIV-positive women originated from sub-Saharan Africa (98.8%).

**Table 1:** Characteristics of asylum seekers who gave birth during their stay in asylum reception in The Netherlands, 2000–2008.

	Number of asylum seekers who gave birth	% of all women
**All women**	4,854	100.0
**Age group at delivery**		
13–19 ^a^	831	17.1
20–29	2,654	54.7
30–49	1,369	28.2
**Family composition**		
Woman who arrived with partner ^b^	1,178	36.6
Woman who arrived without partner ^b^	2,250	46.4
Unaccompanied minor asylum seeker	826	17.0
**Region of origin** ^c^		
Sub-Saharan Africa	2,308	47.5
Europe and Central Asia	871	17.9
Middle East and North Africa	753	15.5
South Asia	504	10.4
East Asia and Pacific	255	5.3
Latin America and Caribbean	20	0.4
Stateless and unknown	143	2.9
**Migration phase at time of conception**		
Before arrival in The Netherlands	2,028	41.8
Within 1 year after arrival	1,267	26.1
As from 1 year after arrival	1,559	32.1
**HIV testing policy at first antenatal consultation**		
Selective	3,145	64.8
Universal	1,709	35.2

^a^ Of the girls in this age group 224 were 19 years of age, 263 were 18 years, 222 were 16 years, 62 were 15 years, 14 were 14 years, and 1 was 13 years;

^b^ Excluding UMAs;

^c^ Countries of origin of the women from Sub-Saharan Africa are presented in Table 2; main countries of origin of the women from the other regions of origin were Afghanistan (454 women), Iran (129), Iraq (470), China (242), countries of the former Soviet Union (518) and Syria (96).

The HIV prevalence among the 2,308 women from sub-Saharan Africa was 3.4% and is much higher than the overall antenatal HIV prevalence in The Netherlands (0.04%; OR = 82.5; 95%CI 63.7–106.7) [21]. Among the 2,546 women from other regions of origin, one woman was recorded to be HIV positive (prevalence 0.04%). The prevalence for women from sub-Saharan Africa was high compared to women from all other regions of origin (OR = 90.2; 95%CI 12.5–648.8). The antenatal HIV prevalence among women from all other regions of origin was similar to the overall antenatal HIV prevalence in The Netherlands (OR = 0.92; 95%CI 0.13–6.52). However, the wide 95%CI implies that precise estimates of HIV prevalence rates cannot be made for this group.

Because of the concentration of cases in sub-Saharan African women, further analyses were restricted to this group (Table 2). The HIV prevalence tended to be higher among women aged 13–19 and 30–49 years than among women aged 20–29. The prevalence was higher among women who arrived in reception without partner (3.4%) and among UMAs (4.5%) compared to women who arrived with partner (1.7%). However, the 95%CI strongly overlapped for age group as well as family composition, implying that the observed differences could not be demonstrated with statistical significance (Table 2).

**Table 2:** HIV prevalence among women from sub-Saharan Africa and association with demographic and pregnancy related variables.

	Number of HIV-positive women	Total number of women	HIV prevalence (%)	Odds ratio ^a^	95%CI ^b^
**Age group at delivery** ^c^					
13–19	24	551	4.4	1	-
20–29	37	1,308	2.8	0.89	0.36–2.18
30–49	18	449	4.0	1.36	0.47–3.95
**Family composition**					
Woman who arrived with partner ^d^	6	347	1.7	1	-
Woman who arrived without partner ^d^	46	1,367	3.4	1.82	0.75–4.44
Unaccompanied minor asylum seeker	27	594	4.5	2.59	0.79–8.49
**Country of origin**					
Angola	9	497	1.8	1	-
Burundi	9	108	8.3	4.58	1.70–12.30
Cameroon	5	38	13.2	7.63	2.40–24.28
Democratic Republic of Congo	8	254	3.1	1.64	0.62–4.34
Eritrea	1	44	2.3	1.19	0.15–9.81
Guinea-Conakry	6	154	3.9	1.76	0.60–5.21
Ivory Coast	4	51	7.8	3.65	1.04–12.79
Liberia	4	69	5.8	3.04	0.89–10.42
Nigeria	1	87	1.1	0.60	0.08–4.88
Rwanda	8	47	17.0	11.84	4.25–32.95
Sierra Leone	10	257	3.9	1.95	0.77–4.93
Somalia	7	401	1.7	0.94	0.33–2.66
Sudan	3	180	1.7	0.99	0.26–3.74
Togo	2	49	4.1	1.86	0.38–9.02
Other sub-Saharan countries ^e^	2	72	2.8	1.42	0.30–6.79
**Migration phase at time of conception**					
Before arrival in The Netherlands	34	1,056	3.2	1	-
Within one year after arrival	22	588	3.7	1.15	0.66–2.01
As from one year after arrival	23	664	3.5	0.96	0.52–1.77
**HIV testing policy at first antenatal consultation**					
Selective	50	1,506	3.3	1	-
Universal	29	802	3.6	1.13	0.66–1.93

^a^ Based on multivariate model including all variables in this table;

^b^ 95% Confidence intervals;

^c^ Based on multivariate model including all variables in this table,

^d^ Excluding UMAs,

^e^ Includes women from: Chad, Comoros, Gambia, Ghana, Kenya, Mali, Mauretania, Namibia, Niger, Senegal, Uganda, Western Sahara and South Africa

The HIV prevalence for women from sub-Saharan Africa ranged from 1.1% for Nigerian women to 17.0% for Rwandan women (Table 2). The multivariate analysis shows that differences between the countries cannot be explained by the other variables in the model (Table 2). Only small differences in the HIV prevalence rate were observed between women who were pregnant at arrival, women who got pregnant within one year after arrival, and women who got pregnant as from one year after arrival (Table 2). The HIV prevalence increased from 3.3% before the implementation of universal HIV testing to 3.6% afterwards. After correction for the demographic variables, the 95%CI overlapped, implying that this small increase was not statistically significant (Table 2).

Among the 384 sub-Saharan African women who gave birth to more than one child during their stay in reception, 13 were HIV positive at the last delivery in reception (3.4%). Four of these women were not recorded to be HIV positive at their first pregnancy in reception and were probably infected with HIV during their stay in reception. Two of these women arrived in reception without a partner; both women were younger than 30 years. The two other women were UMA’s. In addition, one woman who was not recorded to be HIV positive during her last pregnancy in reception was recorded to be HIV positive a few years later.

Six of the children born to an HIV-positive mother were recorded to be HIV positive at birth. They were all born before the implementation of the universal antenatal HIV screening in July 2004. During this period of the study 62 children were born to an HIV positive mother; the MTCT rate was 9.8%.

Among UMAs the highest prevalence rates were found for girls from the Democratic Republic of Congo, Sierra Leone and ‘Other countries’ (Table 3). The highest prevalence was found among the UMAs who got pregnant in the first year after arrival in The Netherlands (Table 3). The prevalence in this group (8.3%) was three times higher than in UMAs who were pregnant at arrival (95%CI 1.17–7.75).

**Table 3:** HIV prevalence among unaccompanied minor asylum seekers (UMAs) aged 13–18 years from sub-Saharan Africa and association with demographic and pregnancy related variables.

	Number of HIV-UMAs	Number of UMAs	HIV prevalence (%)	Odds ratio ^a^	95%CI ^b^
**Country of origin**					
Angola	2	130	1.5	1	-
Democratic Republic of Congo	4	56	7.1	5.22	0.92–29.68
Guinea-Conakry	2	97	2.1	1.36	0.19–10.03
Sierra Leone	6	107	5.6	3.72	0.73–18.97
Other countries ^c^	13	204	6.4	4.59	1.00–20.99
**Migration phase at time of conception**					
Before arrival in The Netherlands	8	260	3.1	1	-
Within one year after arrival	11	132	8.3	3.00	1.17–7.75
As from one year after arrival	8	202	4.0	1.50	0.52–4.38
**HIV testing policy at first antenatal consultation**					
Selective	20	401	5.0	1	-
Universal	7	193	3.6	0.68	0.26–1.83

^a^ Based on multivariate model including all variables in this table;

^b^ 95% Confidence intervals;

^c^ Includes UMAs from: Benin, Burundi, Cameroon, Comoros, Gambia, Ivory Coast, Liberia, Niger, Nigeria, Rwanda, Sudan, Togo and Uganda.

## Discussion

The prevalence of HIV among women from sub-Saharan Africa who gave birth during their stay in asylum reception was 3.4%. Among women from all other regions together, the prevalence was 0.04%. Of all women who were HIV positive, 98.8% originated from sub-Sahara Africa. The highest HIV prevalence rates among sub-Saharan African women were found among women who arrived in reception without partner and especially UMAs. The prevalence of HIV ranged from less than 2% for women from Nigeria, Somalia and Sudan to more than 10% for women from Cameroon and Rwanda.

### Strengths and limitations

Strengths of this study are the large number of women in the study, the nationwide coverage, the availability and completeness of demographic data for all women who delivered in asylum reception in The Netherlands during the study period and their newborns, and the availability of data under a policy of universal antenatal HIV testing during the second half of the study.

This study also has certain limitations. First of all, despite the large study population, the numbers of HIV cases in subgroups (e.g. by country of origin) are small. Therefore, the prevalence rates estimated for these specific groups lack precision. Furthermore, the absence of data for pregnancies that were not carried to term may have influenced HIV prevalence rates, as HIV may be associated with spontaneous and induced abortions [26,27].

HIV prevalence rates among women attending antenatal care are generally considered to be a proxy for the HIV prevalence in the general population. However, it is unclear whether this also applies to the asylum population [28,29]. Many factors may influence the variations in HIV prevalence rates between pregnant women, other women and men [30]. Women may be particularly vulnerable to HIV infection in times of war and during flight [12,31]. In addition, asylum seekers are relatively young and more often male and single than the general population. Due to lack of detailed knowledge on such factors, it is unknown whether the HIV prevalence rates found among the asylum seekers who gave birth in The Netherlands are higher or lower than in the overall asylum-seeking population.

In addition, the recording of HIV diagnoses on the medical problem list may have been incomplete because results from antenatal screening may not always have been communicated to MOA or the family physician. This may have resulted in underestimation of HIV prevalence rates.

Finally, the change in the HIV testing policy in July 2004 from selective to opting-out screening may have influenced the results. In the first part of the study period, HIV cases may have been missed and as a consequence the HIV prevalence rates may be underestimated. However, the accounts of health professionals suggest that the uptake of HIV testing among pregnant asylum seekers, especially from sub-Sahara Africa, was already very high before 2004. In addition, the multivariate model showed no substantial or significant increase in HIV prevalence rates after the introduction of universal testing compared to the previous period of selective testing.

With respect to the generalisability of our findings to other host countries, it has to be taken into account that the composition of the asylum population may strongly differ between countries. In addition, the HIV prevalence may be influenced by differences between host countries in reception conditions, HIV screening policies and the availability of preventive interventions for asylum seekers [32,33].

### Interpretation of results

Comparisons to other countries can be made only with regards to HIV prevalence in the general asylum population, as antenatal surveillance data are not available for asylum seekers in other host countries. The antenatal HIV prevalence in this study (1.6%) is low compared to the prevalence rate found in asylum-seeking men and women in a small study in the UK (3.9%) and comparable to the prevalence rate observed in Italy (1.5%) [13]. The HIV prevalence among sub-Saharan African women who delivered in reception in our study (3.4%) was higher than among the sub-Saharan African asylum-seeking women who were tested for HIV at arrival in the United States (1.4%) [34]. The difference might be related to the composition of the study population (women who gave birth in The Netherlands compared to all women at arrival in the US) or to a different distribution of the countries of origin. However, the US study does not provide data on country of origin.

The HIV prevalence rates that we observed for sub-Saharan women are similar or higher than the rates observed among sub-Saharan African women living in their countries of origin or in refugee camps (S1 Table). Differences may be explained by various factors, e.g. declining HIV prevalence rates in the countries of origin and selection on HIV risk during the migration processes. The higher HIV rates found among Rwandan asylum seekers compared to rates in Rwanda, for example, may reflect the strong decrease in HIV prevalence observed in Rwanda between 1998 and 2003 [35]. In addition, urban-rural differences in HIV prevalence may have played a role: in 2002 the antenatal HIV prevalence was 13% in Kigali whereas it was 3% in rural settings [35].

For regions of origin other than sub-Saharan Africa, the antenatal HIV prevalence rate was at the same level as in the Dutch population. The number of women in the study was too small to obtain precise estimates. However, for the countries of origin from which most of the asylum seekers originated (Afghanistan, Iran, Iraq, and Syria), the HIV prevalence estimates for the population aged 15–49 years in 2006 were 0.2% or less, which is similar to or lower than our estimates for those in The Netherlands (0.2%) [36]. Although the estimated HIV prevalence in the Russian Federation was higher (1.1%), no cases were observed in Russian women who delivered in reception in our study [36].

The higher HIV prevalence rates among UMAs and women who arrived without partner may reflect the particular vulnerability of these girls and women, both in their countries of origin, during the flight and in the host country [12,37]. Our finding that HIV prevalence rates among women who got pregnant after arrival in The Netherlands were as high as among women who were already pregnant at arrival, may reflect the absence of an active HIV screening policy for all men and women. An active HIV screening policy would have made more women aware of their HIV infection, so that they could choose not to have unprotected sex or to postpone becoming pregnant. The high HIV prevalence rates among UMAs who got pregnant within one year after arrival, may reflect the particular vulnerability of these girls shortly after arrival [12,38]. We documented a number of cases showing transmission of HIV to asylum-seeking women and girls during their stay in asylum reception in the Netherlands. This adds to the evidence pointing towards HIV transmission after migration to Europe and underlines the importance of preventive interventions [38].

The decrease in the MTCT rate from 9.8% before the implementation of universal HIV testing to 0% after implementation in 2004, illustrates the potential effectiveness of this screening policy with respect to the transmission from mother to child in the asylum population. This is in line with the effectiveness shown for the general population [21].

## Conclusions

The HIV prevalence among asylum seekers from sub-Saharan Africa who gave birth in The Netherlands is high compared to the host population in The Netherlands. The prevalence differs between countries of origin. Higher HIV prevalence rates were observed among sub-Saharan African UMAs and women who arrived without a partner compared to women who arrived with partner. For women from other regions than sub-Saharan Africa, the HIV prevalence rate did not differ from the overall pregnant population in The Netherlands.

The results of this study underline the importance of offering a voluntary HIV test to newly arriving asylum seekers from sub-Sahara Africa [28,39,40]. In addition, preventive interventions aimed at improving sexual and reproductive health knowledge and skills and reducing HIV stigma should be offered to all asylum seekers. Several studies and experiences of health professionals show that asylum seekers need and want such information and training [40–45]. As the risk of unprotected sex seems to be high in the first year in the host country, especially among UMAs and women who arrived in the host country without partner, preventive sexual and reproductive health interventions should be available and accessible to them as from arrival in the host country [38,40]. This is important for the health of individual asylum seekers as well as for the prevention of HIV transmission at population level. Finally, continuous systematic collection of epidemiological data is recommended as the HIV-epidemiology among asylum-seekers is expected to change over time.

## Supporting Information

S1 Table Comparison of antenatal HIV prevalence rates between asylum seekers who gave birth in The Netherlands and antenatal prevalence rates in countries of origin and refugee camps in the region of origin. (DOCX)
