# Supplementary material for: High HIV Prevalence among Asylum Seekers Who Gave Birth in the Netherlands: A Nationwide Study Based on Antenatal HIV Tests
Source: PLoS One. 2015 Aug 21;10(8):e0134724. doi: 10.1371/journal.pone.0134724 (PMC4546638; doi:10.1371/journal.pone.0134724)
Supplement: S1 Table — (DOCX) [file pone.0134724.s001.docx]

**S1 Table. Comparison of antenatal HIV prevalence rates between asylum seekers who gave birth in the Netherlands and antenatal prevalence rates in countries of origin and refugee camps in the region of origin.**

|  | **Antenatal HIV prevalence (%)** | | |
| --- | --- | --- | --- |
| **Country of origin** | **Asylum seekers in the Netherlands**  **(this study)** | **WHO data for country of origin**  **2003-2004[1]** | **Data for refugees in refugee camps in the region[2]** |
| Rwanda | 17.0 | 4.6 | - |
| Cameroon | 13.2 | [5.4] ^a^ | - |
| Burundi | 8.3 | 4.8 | 1.6 – 4.8 |
| Ivory Coast | 7.8 | 8.3 | - |
| Liberia | 5.8 | [2.0-5.0] ^a^ | - |
| Togo | 4.1 | 3.6 | - |
| Guinea-Conakry | 3.9 | 4.2 | - |
| Sierra Leone | 3.9 | 3.0 | - |
| DR Congo | 3.1 | 4.2 | 1.0 - 6.7 |
| Eritrea | 2.3 | 1.8 | 4.1 |
| Angola | 1.8 | 2.4 | - |
| Sudan | 1.7 | [1.6] ^a^ | 0.8 – 5.0 |
| Somalia | 1.7 | [0.9] ^a^ | 0.6 – 1.4 |
| Nigeria | 1.1 | 4.3 | - |

^a^ Estimates for adult population 15-49 years by UNAIDS[3]

Reference List

1. World Health Organization Regional Office for Africa: HIV/AIDS Epidemiological Surveillance Report for the WHO African Region 2005 Update*.* Harare; 2005. Available: <http://www.who.int/hiv/pub/surveillance/hivinafrica2005e_web.pdf>.

2. Spiegel PB, Bennedsen AR, Claass J, Bruns L, Patterson N, Yiweza D, Schilperoord M: Prevalence of HIV infection in conflict-affected and displaced people in seven sub-Saharan African countries: a systematic review**.** Lancet 2007; 369:2187-2195.

3. UNAIDS: 2006 Report on the global AIDS epidemic; a UNAIDS 10th anniversary special edition*.* Geneva; 2006. Available: <http://data.unaids.org/pub/report/2006/2006_gr_en.pdf>.
